# Abnormal sleep duration is associated with sarcopenia in older Chinese people: A large retrospective cross-sectional study

**DOI:** 10.1515/med-2024-0938

**Published:** 2024-04-01

**Authors:** Xilin Peng, Ruihao Zhou, Congqi Liu, Xudong Chen, Tao Zhu, Guo Chen

**Affiliations:** Department of Anesthesiology, National Clinical Research Center for Geriatrics, West China Hospital, Sichuan University, Chengdu, Sichuan 610041, China; The Research Units of West China (2018RU012)-Chinese Academy of Medical Sciences, West China Hospital, Sichuan University, Chengdu, China; Department of Anesthesiology, National Clinical Research Center for Geriatrics, West China Hospital, Sichuan University, No. 37 Guoxue Xiang, Wuhou District, Chengdu, Sichuan 610041, China

**Keywords:** sarcopenia, sleep duration, possible sarcopenia, older Chinese people

## Abstract

**Aim:**

Abnormalities in sleep patterns are a common health problem for the older adults. The relationship between sarcopenia and sleep duration in older people is controversial. This research is to examine the association between sleep duration and sarcopenia.

**Methods:**

We drew 21,095 adults from the China Health and Retirement Longitudinal Survey (CHARLS). Not only we explore the relationship between sleep duration and sarcopenia, but also compare sleep duration to three sarcopenia subcomponents. Moreover, the sensitivity analysis was conducted by the gender and residence area to ascertain the discrepancy, separately. Finally, using restricted cubic spline to find the non-linear association between them.

**Results:**

Among 7,342 community older adults engaged by CHARLS in 2015, the incidence of possible sarcopenia and sarcopenia was 23.14 and 11.30%, separately. Sleep duration (≤6 h) [OR(95%CI) = 1.30(1.03–1.65), *p* < 0.05] and (≥8 h) [OR(95%CI) = 1.33(1.05–1.69), *p* < 0.05] were significantly linked with possible sarcopenia, while long sleep duration (≥8 h) [OR(95%CI) = 1.41(1.01–2.02), *p* < 0.05] was correlated strongly with sarcopenia. A non-linear relationship (U-shaped) between sarcopenia risk and sleep duration was found (*p* for non-linear = 0.009).

**Conclusions:**

Our findings highlight the importance of sleep duration in the onset of sarcopenia and might assist older persons to maintain good sleeping habits.

## 1 Introduction

China, which has one-fifth of the world’s elderly and the world’s largest elderly population (≥60 years old), will further increase its aging burden in 2022 when the second baby boom generation (born between 1962 and 1975) begins to retire [1]. By 2050, it is anticipated that the prevalence of sarcopenia among the elderly in China would surpass 500 million [2]. Sarcopenia, defined by the Asian Working Group for Sarcopenia 2019 (AWGS2019), is an age-related illness [3]. Long-term clinical outcomes demonstrated that AWGS-defined sarcopenia was highly related to an elevated risk of physical limits at 4 years, sluggishness at 7 years, and death at 10 years [4]. AWGS2019 discourages the use of the phrase “possible sarcopenia” in medical or research settings. In clinical practice, possible sarcopenia is sufficient to trigger an examination of causes and prepare early intervention.

As a vital human requirement, adults should sleep for 7–8 h every night, according to the National Sleep Foundation’s recommendations. Typical changes in sleep patterns linked with aging include decreased total sleep time and sleep efficiency, which impacted up to 50% of the elderly population [5]. Sleep problems in the elderly not only affect their quality of life but also put them at greater risk of death, too little or too much sleep has been shown to lead to diabetes, metabolic syndrome, obesity, cardiovascular disease, and death [6,7]. In a longitudinal cohort research of sleep duration and muscle loss in Japan, long sleep length was associated with progression to sarcopenia after 4 years, whereas short sleep duration was not [8]. However, in a cross-sectional survey of 1,068 older adults, there was no correlation between sleep duration and sarcopenia [9]. The relationship between total sleep duration and sarcopenia remains debatable. The AWGS2019 definition of “possible sarcopenia” was validated, and its diagnostic accuracy for sarcopenia was found to be remarkable [10].

Based on our current search, there are few unanimous results regarding the association between sleep duration and sarcopenia, especially in community-dwelling older adults in China. To offset the potential economic and social effects of China’s aging population, this study investigated the association between sleep duration and sarcopenia and possible sarcopenia.

## 2 Methods

### 2.1 Study population

The CHARLS is a micro-database that is nationally representative. The preliminary survey yielded high-quality survey data. A face-to-face computer-assisted personal interview comprising physical measures, blood sample collection, and other evaluations were employed in the CHARLS. The Peking University Biomedical Ethics Review Committee (IRB00001052-11015) accepted the study design and methodology and all subjects provided informed consent. More information is available on the CHARLS project website (http://charls.pku.edu.cn/).

For this retrospective cross-sectional study, we selected 21,095 people from 2015, the inclusion criteria of participants must meet the requirements of true age (≥60 years), gender, and physical examination related data such as height, weight, grip strength, 5 chair standing times (5-TCS), sleep duration, etc.

### 2.2 Total sleep duration

The CHARLS study queried participants about their sleep durations throughout the previous month using a questionnaire and data collection. An earlier comprehensive review and meta-analysis identified a nonlinear U-shaped connection between sleep duration and the relative risk of developing sarcopenia, with nadirs between 6 and 8 h per night [11]. We classified total sleep duration into three groups: short (≤6 h), normal (6–8 h), or long (≥8 h).

### 2.3 Sarcopenia

The AWGS2019 agreement maintains a maximum age restriction of 60 or 65 years, depending on how each nation defines “elderly.” According to consensus recommendations, three measurements are required to diagnose sarcopenia: muscle strength, muscle mass, and physical performance [3].

The handgrip strength, which was assessed in kilograms (kg) by having the subject squeeze a dynamometer as firmly as possible, represents muscle strength. Everyone was tested twice with both hands, the dynamometer held at a right angle, and the handle squeezed for several seconds. The greatest value of a single measurement is taken, whether it is with the dominant hand, the left and right hands, or both. Normal minimum handgrip strength according to the AWGS2019 was 28 kg for males and 18 kg for females.

Muscle mass, using an algorithm previously verified in a Chinese population, the appendicular skeletal muscle mass (ASM) was used to measure muscle mass: ASM 0.193 × Body Weight (Kilogram) + 0.107 × Height (Centimeter) - 4.157 * Sex (Men = 1; Women = 2) - 0.037 × Age (Year) - 2.63118

The skeletal muscle mass index (SMI) was determined by dividing the ASM by the square of the individual’s height in meters (SMI = ASM/height^2^). Numerous investigations have demonstrated that SMI and dual-energy X-ray absorptiometry (DXA) are in good agreement [12,13]. The cutoff for deficient muscle mass was based on the lowest 20% of SMI for the research population. In our study, the cutoff values were 4.93 kg/m^2^ for women and 6.78 kg/m^2^ for men.

Based on the recommendations of AWGS2019, meanwhile in combination with the physical examination items of CHARLS, 5-CST is used as the data of the physical fitness test. The participants were asked to maintain their arms crossed over their chest, stand up straight, and sit down as swiftly as possible five times, without hesitating or using their arms to propel themselves. If the participants used their arms or others during the recording of the time, the test would be considered unsuccessful. Low physical performance was characterized by a 5-CST of more than 12 s.

Possible sarcopenia is described by reduced muscular strength or poor physical performance. Sarcopenia is distinguished by reduced muscle mass and strength, or a combination of poor physical performance. In addition, people with severe sarcopenia have low muscular mass, limited muscle strength, and poor physical performance. Since there are only 165 (3.62%) people defined as having severe sarcopenia, they are placed in the sarcopenia group. All subjects were then split into three groups: non-sarcopenia, possibly sarcopenia, and sarcopenia.

### 2.4 Covariates

Demographic data such as age, gender, degree of education, marital status, and dwelling area, as well as socioeconomic groupings [14]. Behavioral characteristics include smoking status, number of days of alcohol consumption in the past year, activities of daily living (ADL), and instrumental activities of daily living (IADL). ADL and IADL were divided into have no difficulty group (did not need help in all ADL or IADL items) and have difficulty group (needed help in any ADL or IADL items) [15]. Physical examination including body mass index (BMI according to the WHO definition of China <18.5, 18.5–24.0, 24.0–28.0, and ≥28.0 kg/m^2^) [16], self-reported 14 physician-diagnosed comorbidities (hypertension, dyslipidemia, diabetes, cancers, chronic lung diseases, liver diseases, heart diseases, stroke, kidney diseases, digestive diseases, psychiatric diseases, memory-related disease, arthritis or rheumatism, and asthma) were divided into three groups: none of these, only one disease or have two or more diseases, hypertension (self-reported, medication, or systolic blood pressure ≥140 mmHg or diastolic blood pressure ≥90 mmHg) [17], diabetes (self-reported, medication, fasting plasma glucose ≥126 mg/dL, or non-fasting plasma glucose ≥200 mg/dL), chronic kidney disease (eGFR <90 mL/min is renal insufficiency, glomerular filtration rate estimated by Cockcroft-Gault equation) [18], cognitive function score (The total cognitive score was calculated by adding the scores for orienting [5 points], calculation [5 points], recollection [20 points], and drawing [1 point], for a total of 31 points.), depression symptoms assessments (depressed disorders were measured using the 10-item short form of the Center for Epidemiologic Studies Depression Scale [CES-D-10]), and knee height.

### 2.5 Statistical analysis

Continuous variables with normal distributions were represented by mean and standard deviation (SD), while those with abnormal distributions were represented by medians and interquartile ranges. For categorical variables, percentages were employed. To compare participant characteristics by total sleep time category, one-way ANOVA, the Kruskal–Wallis *H*-test, and the Chi-square test were utilized.

The relationship between sarcopenia and total sleep duration was examined using unadjusted and adjusted logistic regression models. Pre-set models were used to guarantee the reliability of the findings. Besides, the sensitivity analysis was run independently for gender and residential area to determine the disparity. Restricted cubic spline (RCS) with four knots and a logistic regression model were used to analyze the nonlinear association between sleep time (continuous variables) and sarcopenia (bicategorical variables) and to investigate the dose–response relationship. The model was adjusted to account for age, gender, marital status, drinking, ADL, IADL, co-morbidities, and CES-D-10 scores. R 4.2.2 was used to complete RCS and optimum cutoff points with the “ggrcs” package [19].

With 95% confidence intervals (95%CI), the outcomes of the regression analysis were presented as odds ratio (OR). The level of statistical significance was established at *p*-value <0.05 and two-sided tests. All statistical analysis was employed with Stata/MP 17.0 (Stata Corporation, College Station, USA).

## 3 Results

### 3.1 Description of the study population

We picked 7,342 individuals from 2015 in CHARLS (Figure S1). Table 1 depicts a variety of participant characteristics according to the duration of sleep. With a mean age of 67.89 ± 6.38 years, 50.04% of them were female, 74.17% of whom lived in rural areas. Possible sarcopenia accounted for 23.14% (1,699/7,342), whereas sarcopenia accounted for 11.30% (830/7,342) of the total participants.

**Table 1:** Characteristics of participants according to total sleep duration in CHARLS 2015

Characteristic	Total *N* = 7,342	Short *N* = 2,844	Medium *N* = 1,560	Long *N* = 2,938	*P*-value
Age (years, M ± SD)	67.89(6.38)	68.36(6.63)	67.15(5.93)	67.81(6.33)	<0.001
Female	50.04(3,674)	57.81(1,644)	46.09(719)	44.62(1,311)	<0.001
**Education degree**					
Illiteracy	31.88(2,119)	35.99(925)	24.27(341)	31.94(853)	<0.001
Primary school	46.69(3,103)	45.60(1,172)	48.40(680)	46.84(1,251)	
Middle school	19.88(1,321)	17.04(438)	25.20(354)	19.81(529)	
University and above	1.55(103)	1.36(35)	2.14(30)	1.42(38)	
**Marital status**					
Married/cohabitation	77.88(5,718)	74.05(2,106)	81.41(1,270)	79.71(2,342)	<0.001
Single/divorced/bereave	22.12(1,624)	25.95(738)	18.59(290)	20.29(596)	
**Residential area**					
Urban	25.83(1,892)	24.76(702)	33.80(526)	22.64(664)	<0.001
Rural	74.17(5,432)	75.24(2,133)	66.20(1,030)	77.36(2,269)	
**Socioeconomic status (CNY)**					
0–4,283	2,392(1,164)	24.78(452)	20.63(215)	24.84(497)	0.002
4,284–9,550	33.86(1,648)	34.76(634)	32.44(338)	33.78(676)	
9,551 or more	42.22(2,055)	40.46(738)	46.93(489)	41.38(828)	
**Smoke**					
Never	54.78(3,891)	59.29(1,640)	52.51(795)	51.58(1,456)	<0.001
Current	32.44(2,304)	28.52(789)	33.69(510)	35.60(1,005)	
Ever but quit	12.78(908）	12.18(337)	13.80(209)	12.82(362)	
**Drink**					
Never	25.90(1,901)	24.00(682)	21.19(360)	26.99(793)	<0.001
Current	7.47(548)	6.86(195)	7.06(120)	7.35(216)	
Ever but quit	66.63(4,890)	69.14(1,965)	71.15(1,219)	65.66(1,929)	
Difficulty in daily activities	30.92(1,710)	27.48(308)	37.26(863)	25.74(539)	<0.001
Difficulty in instrumental activities	36.80(2,702)	44.20(1,257)	29.49(460)	33.53(985)	<0.001
**BMI (kg/m** ^**2**^ **, M ± SD)**					
<18.5	7.75(569)	8.61(245)	6.86(107)	7.39(217)	<0.001
18.5 ≤ BMI < 24.0	51.80(3,803)	54.40(1,547)	48.78(761)	50.88(1,495)	
24.0 ≤ BMI < 28.0	29.86(2,192)	27.00(768)	32.63(509)	31.14(915)	
≥28	10.60(778)	9.99(284)	11.73(183)	10.59(311)	
**Number of co-morbidities**					
0	49.02(3,599)	46.17(1,313)	53.21(830)	49.56(1,456)	<0.001
1	6.96(511)	6.36(181)	6.35(99)	7.86(231)	
≥2	4.02(3,232)	47.47(1,350)	40.45(631)	42.58(1,251)	
Hypertension	27.84(2,044)	29.29(833)	25.00(390)	27.94(821)	0.010
Diabetes	11.65(855)	12.27(349)	10.71(167)	11.54(339)	0.293
Chronic kidney disease	47.67(3,500)	51.09(1,453)	43.27(675)	46.70(1,372)	<0.001
Cognitive assessment (score, M ± SD)	12.06(5.98)	11.34(6.00)	13.59(5.81)	11.95(5.90)	<0.001
CES-D-10 items (score, M ± SD)	8.28(6.49)	10.26(7.00)	7.25(5.93)	6.96(5.76)	<0.001
Knee height (cm, M ± SD)	47.40(3.68)	46.96(3.52)	44.71(3.75)	47.66(3.74)	<0.001
**Sarcopenia defined on AWGS 2019**					
Without sarcopenia	65.55(4,831)	61.53(1,750)	71.96(1,121)	66.10(1,942)	<0.001
Possible sarcopenia	23.14(1,699)	25.00(711)	19.94(311)	23.04(677)	
Sarcopenia	11.30(830)	13.47(383)	8.21(128)	10.86(319)	
**Components of sarcopenia**					
Low muscle mass	44.24(3,248)	36.99(1,052)	49.68(775)	48.37(1,421)	<0.001
Low muscle strength	17.46(1,282)	19.23(547)	14.17(221)	17.49(514)	<0.001
Low physical performance	24.79(1,820)	28.62(814)	19.29(301)	24.00(705)	<0.001

Abbreviations: AWGS 2019, Asian Working Group for Sarcopenia 2019; BMI, body mass index; CES-D-10, 10-item short form of the Center for Epidemiologic Studies Depression Scale; CNY, Chinese Yuan.

### 3.2 Association between total sleep duration and possible sarcopenia

In the overall research population, the crude model (Model 1) revealed a substantial connection between short or long total sleep time and the possibility of sarcopenia, for short total sleep duration (≤6 h) [OR(95%CI) = 1.46(1.26–1.71), *p* < 0.001] and for long total sleep duration (≥8 h) [OR(95%CI) = 1.26(1.08–1.46), *p* < 0.001]. Model 4 revealed that the short sleep duration group [OR(95%CI) = 1.30(1.03–1.65), *p* < 0.05] and the long sleep duration group [OR(95%CI) = 1.33(1.05–1.69), *p* < 0.05] were substantially related with a greater probability of sarcopenia compared to the typical sleep length groups (6–8 h) (Table 2).

**Table 2:** Associations between sarcopenia and total sleep duration by multinomial logistic regression model

Total sleep duration	Possible sarcopenia	Sarcopenia	Possible sarcopenia	Sarcopenia
	OR (95%CI)	OR (95%CI)	OR (95%CI)	OR (95%CI)
	**Model 1** ^**a**^		**Model 2** ^**b**^	
Short	1.46(1.26–1.71)***	1.92(1.55–2.37)***	1.44(1.18–1.76)***	1.46(1.08–1.96)**
Medium	Ref.	Ref.	Ref.	Ref.
Long	1.26(1.08–1.46)***	1.44(1.16–1.79)***	1.28(1.05–1.55)**	1.41(1.04–1.90)**
	**Model 3** ^**c**^		**Model 4** ^**d**^	
Short	1.42(1.16–1.74)***	1.43(1.06–1.93)**	1.30(1.03–1.65)**	1.13(0.80–1.62)
Medium	Ref.	Ref.	Ref.	Ref.
Long	1.24(1.02–1.52)**	1.39(1.03–1.88)**	1.33(1.05–1.69)**	1.41(1.01–2.02)**

***: *p*-value < 0.001, **: *p*-value < 0.05. ^a^Crude model. ^b^Adjusted for age, gender, marital status, residential area, socioeconomic status. ^c^Adjusted for age, gender, marital status, residential area, socioeconomic status, smoke, drink. ^d^Adjusted for age, gender, marital status, residential area, socioeconomic status, smoke, drink, difficulty in daily activities, difficulty in instrumental activities, BMI, co-morbidities, hypertension, diabetes, chronic kidney disease, cognitive assessment scores, CES-D-10 items scores.

Ref, reference; CI, confidence interval; OR, odds ratio.

### 3.3 Association between total sleep duration and sarcopenia

The outcomes of several models produced from multiple logistic regression studies are presented in Table 2. Compared with normal sleeping for 6–8 h, short total sleep duration (≤6 h) had a nearly two-fold increased likelihood of sarcopenia [OR(95%CI) = 1.92(1.55–2.37), *p* < 0.001], as for long total sleep duration (≥8 h) had an over one-fold increased likelihood of sarcopenia [OR(95%CI) = 1.44(1.16–1.79), *p* < 0.001]. After adjusting all relative factors (Model 4), the risk of the long group [OR(95%CI) = 1.41(1.01–2.02), *p* < 0.05] was higher than the control group, and no significant result was displayed in the short group [OR(95%CI) = 1.13(0.80–1.62)].

### 3.4 Association between the length of sleep and the specific sarcopenia components

The short group had a significant relationship with lower physical performance [OR(95%CI) = 1.32(1.05–1.67), *p* < 0.05], as same as the long group [OR(95%CI) = 1.35(1.06–1.71), *p* < 0.05]. Compared with the normal group, lower grip strength was associated with the long total sleep duration [OR(95%CI) = 1.36(1.03–1.79), *p* < 0.05], but not with the shorter total sleep duration. However, the link between total sleep duration and lower SMI was no longer significant (Table 3).

**Table 3:** Associations between development of sarcopenia subcomponents and total sleep duration by logistic regression model

Total sleep duration	Low muscle mass	Low physical performance	Low muscle strength
	OR (95%CI)	OR (95%CI)	OR (95%CI)
Short	1.12(0.73–1.71)	1.32(1.05–1.67)**	1.08(0.82–1.43)
Medium	Ref.	Ref.	Ref.
Long	1.21(0.80–1.83)	1.35(1.06–1.71)**	1.36(1.03–1.79)**

***: *p*-value < 0.001, **: *p*-value < 0.05.

Ref, reference; CI, confidence interval; OR, odds ratio.

### 3.5 Differences in the sexual influence of sleep time on the incidence of sarcopenia

After adjusted factors (Model 4) (Table 4), the association between the short sleep group and possible sarcopenia [OR(95%CI) = 1.11(0.79–1.57), *p* = 0.542] or sarcopenia [OR(95%CI) = 1.36(0.67–2.84), *p* = 0.406] was no longer significant, the long sleep group [OR(95%CI) = 1.47(1.06–2.04), *p* < 0.05] increased the risk of possible sarcopenia, sarcopenia [OR(95%CI) = 1.72(0.83–3.57), *p* = 0.142] which was not significant for elderly male. As for the elderly female, the relationship between the short sleep group and possible sarcopenia [OR(95%CI) = 1.49(1.04–2.12), *p* < 0.05] was significant, sarcopenia [OR(95%CI) = 1.16(0.80–1.68), *p* = 0.446] was not obvious. The risk of possible sarcopenia [OR(95%CI) = 1.06(0.70–1.61), *p* = 0.791] and sarcopenia [OR(95%CI) = 1.32(0.86–2.03), *p* = 0.204] in the long sleep group were not significant.

**Table 4:** Associations between the sexual difference of sarcopenia and total sleep duration by logistic regression models

Outcome variable	Analytic model	Female			Male		
		Short	Medium	Long	Short	Medium	Long
		OR (95%CI)		OR (95%CI)	OR (95%CI)		OR (95%CI)
Possible sarcopenia	Model 1^a^	1.52(1.21–1.90)***	Ref.	1.19(0.94–1.50)	1.42(1.15–1.76)***	Ref.	1.30(1.07–1.60)**
	Model 2^b^	1.55(1.15–2.09)**	Ref.	1.20(0.88–1.65)	1.34(1.02–1.76)**	Ref.	1.31(1.02–1.69)**
	Model 3^c^	1.57(1.16–2.12)**	Ref.	1.18(0.86–1.63)	1.29(0.97–1.70)	Ref.	1.28(0.98–1.66)
	Model 4^d^	1.49(1.04–2.12)**	Ref.	1.16(0.80–1.68)	1.11(0.79–1.57)	Ref.	1.47(1.06–2.04)**
Sarcopenia	Model 1^a^	1.62(1.25–2.10)***	Ref.	1.43(1.09–1.87)**	2.07(1.39–3.06)***	Ref.	1.54(1.04–2.27)**
	Model 2^b^	1.43(1.00–2.04)**	Ref.	1.48(1.03–2.13)**	1.55(0.90–2.65)	Ref.	1.23(0.73–2.10)
	Model 3^c^	1.43(1.00–2.04)	Ref.	1.49(1.03–2.14)**	1.47(0.85–2.53)	ref.	1.14(0.67–1.96)
	Model 4^d^	1.06(0.70–1.61)	Ref.	1.32(0.86–2.03)	1.36(0.66–2.84)	Ref.	1.72(0.83–3.57)

***: *p*-value < 0.001, **: *p*-value < 0.05. ^a^Crude model. ^b^Adjusted for age, gender, marital status, residential area, socioeconomic status. ^c^Adjusted for age, gender, marital status, residential area, socioeconomic status, smoke, drink. ^d^Adjusted for age, gender, marital status, residential area, socioeconomic status, smoke, drink, difficulty in daily activities, difficulty in instrumental activities, BMI, co-morbidities, hypertension, diabetes, chronic kidney disease, cognitive assessment scores, CES-D-10 items scores.

Ref, reference; CI, confidence interval; OR, odds ratio.

### 3.6 Differences in the residential area difference of sleep time on the incidence of sarcopenia

The results showed that there were indeed urban–rural differences in the relationship between sarcopenia and sleep duration (Table S1). The elderly living in urban areas, the risk of possible sarcopenia (short sleep time [OR(95%CI) = 1.24 (0.08–1.92), *p* = 0.330] and long sleep time [OR(95%CI) = 1.25 (0.08–1.95), *p* = 0.334]) and sarcopenia (short sleep time [OR(95%CI) = 1.49(0.66–3.38), *p* = 0.335] and long sleep time [OR(95%CI) = 2.02 (0.85–4.79), *p* = 0.110]) did not show a statistically significant. However, the relatively long [OR(95%CI) = 1.42 (1.06–1.91), *p* < 0.05] or relatively short sleep duration [OR(95%CI) = 1.35(1.00–1.81), *p* < 0.05] of the elderly living in rural areas increased the risk of possible sarcopenia. Short [OR(95%CI) = 1.00(0.66–1.48), *p* = 0.959] and long sleep time [OR(95%CI) = 1.29(0.86–1.93), *p* = 0.214] compared to the normal sleep time prone to have the risk of sarcopenia but no significant.

### 3.7 Non-linear relationship between sleep duration and the risk of sarcopenia

**Figure 1:**
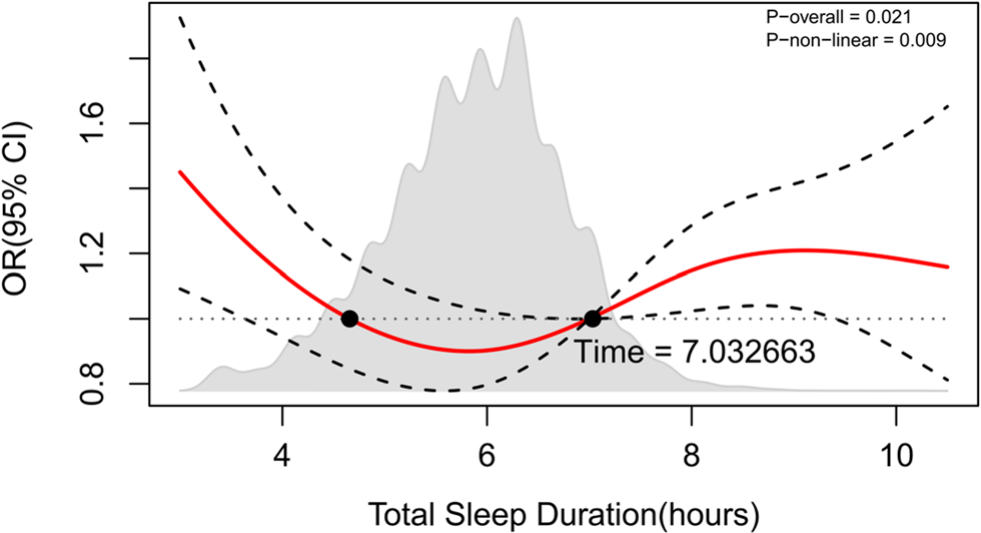
RCS between total sleep duration and the risk of sarcopenia.

Using RCS regression (*p* for non-linear = 0.009), Figure 1 illustrates a non-linear (U-shaped) relationship between sleep duration and the risk of sarcopenia. Participants who slept for an average of 7 h total each day had the lowest risk.

## 4 Discussion

In our present study, we found that sleep disturbance is a risk factor for developing sarcopenia in the Chinese elderly population. The incidence of possible sarcopenia and sarcopenia was 23.14 and 11.30%, respectively. This compares to prior findings indicating that the prevalence of skeletal sarcopenia as defined by the AWGS 2014 ranged from 5.5 to 25.7% [3]. The general frequency of 11–14% among elderly Chinese residents of the neighborhood was constant [20]. The risk factor for possible sarcopenia is an abnormal (≥6 h or ≤8 h) sleep pattern. Since the definition of “possible sarcopenia” is based on the newest recent AWGS2019 recommendations, there were still few recent studies that explored the overall risk factor. The mechanism by which sleep disturbances contribute to possible sarcopenia is reduced muscular strength or physical performance. Referring to restorative theory [21], sleep is necessary for the body’s repair and recovery processes. Many biological reactions develop during sleep including protein synthesis, muscle repairment, and hormone release, which influences muscular strength and physical performance indirectly. Sleep disturbances also have an impact on appetite and hunger [22]. One study stated that two consecutive nights of only 4 h of sleep resulted from leptin decreasing by 18% and growth hormone-releasing peptide increasing by 28%, leading to muscle loss and adipose tissue rising [23].

After additional adjustment for confounders, only long total sleep duration (≥8 h) was a danger factor for the progression of sarcopenia, irrespective of short sleep duration. The finding is in line with the results of other cross-sectional studies [9,24,25]. Furthermore, two longitudinal cohorts of 2 years [26] and 4 years [8] demonstrated that long total sleep duration also increased the incidence of sarcopenia in older adults. This result can be explained by the following mechanisms: prolonged sleep has been linked to the development of insulin resistance (IR), which induces anabolic resistance. Finally, sleep difficulties cause hormonal imbalances promoting the occurrence of sarcopenia [27]. According to the findings of a meta-analysis, high interleukin-6 and C-reactive protein levels were substantially related to longer rather than shorter sleep duration [28]. Some studies [29,30] have found an association between long and short sleep duration and the risk of sarcopenia. This disagreement may be related to discrepancies in research background, sarcopenia diagnostic criteria, and sleep disorder definitions.

We discovered that abnormal sleep duration was associated with poor physical performance and long sleep duration was associated with lower muscle strength, only lower muscle mass was not significantly associated with either sleep duration. These findings were consistent with those of other studies [31,32]. A Japanese study discovered that long sleep (>9 h) was associated with lower physical performance after 4 years of follow-up [8]. Long sleep (>9 h) was linked to poor physical performance (low gait speed) and poor grip strength, but not to low muscle mass. Instead, the results of a community survey [26] showed that it was related to lower muscle mass in addition to lower muscle strength. Various methods of assessing muscle mass can explain this difference. AWGS2019 advises utilizing either multifrequency bioelectrical impedance analysis or DXA both height-adjusted, to measure muscle mass for diagnosing sarcopenia [3]. In terms of gender disparities, the results of our gender stratification are in conflict with those of certain similar studies [24,33]. The elderly are more prone to sarcopenia if they sleep too much or too little. This may cause by the difference in sex hormone secretion, which not only controls muscle growth and development but also modulates sleep rhythm. It is indeed the same as the prior research after doing the RCS analysis [11,33]. Moreover, our findings revealed urban–rural disparities in the link between sarcopenia and sleep duration. This disparity could be attributed to the lifestyles of the elderly living in rural and urban areas. In China, the elderly living in rural areas typically engage in farming activities, and irregular schedules are more common than those living in urban areas. The sleep restorative theory [21] believes that sleep is essential for the process of body repair and rejuvenation. According to the original 2015 CHARLS data, 14,343 (68.42%) of the elderly in the community came from rural regions, while 6,620 (31.58%) were from urban areas. The high concentration of elderly persons in rural areas participating contributes to inequality.

The relationship between sleep and sarcopenia may operate in both directions. Circadian rhythms and molecular clocks play a significant role in the growth and development of skeletal muscle. The Bmal1 (brain and muscle ARNT-like1) gene is one of the key transcriptional activators of biological clock genes [34]. A study [35] discovered that Bmal1 deficiency is associated with aging and causes severe sarcopenia. Besides, abnormal sleep duration causes endocrine disruption (growth hormone, insulin-like growth factor-1, cortisol, testosterone, etc.), blocking myofibril reconstruction, and strengthening in cellular and molecular pathways [36]. Finally, lifestyle behaviors are influenced by sleep patterns. Sleep-deprived individuals are more likely to smoke, lack exercise, consume large amounts of alcohol, and increase sedentary time indirectly [37].

The first advantage of this study is that the sample data we gathered and analyzed were nationally representative. These conclusions may be applied to all of China’s elderly. According to our knowledge, this is the first study to evaluate the association between sleep duration and the risk of sarcopenia in the Chinese elderly using the CHARLS. With the most recent AWGS2019 diagnostic criteria, our findings indicate several potential risk variables for possible sarcopenia, which would help with its early diagnosis and prevention. This study has certain limitations. First, as a cross-sectional study, this research was unable to establish a causal connection between sleep duration and sarcopenia. Additionally, the data on sleep duration are self-reported, which may result in biased findings. The reliability of self-reported sleep duration is also confirmed by the results of several relevant studies based on CHARLS [38–40]. Future research may utilize polysomnography and actigraphy to measure sleep duration [41]. However, we did not investigate the effect of sleep quality in our study because CHARLS lacks this variable. Further studies are required to collect more detailed information to elucidate the association between sleep-related variables and the risk of possible sarcopenia and sarcopenia.

In conclusion, this study found that total sleep duration is non-linearly associated with the risk of developing sarcopenia in older Chinese community residents. Our findings advocate for a greater focus on sleep patterns in the elderly, further additional in-depth investigations to investigate the underlying molecular pathways connecting them.

## Abbreviations

ADL: activities of daily living
ASM: appendicular skeletal muscle mass
AWGS2019: Asian Working Group for Sarcopenia in 2019
Bmal1: brain and muscle ARNT-like 1
BMI: body mass index
CES-D-10: 10-item short form of the Center for Epidemiologic Studies Depression Scale
CHARLS: China Health and Retirement Longitudinal Study
DXA: dual-energy X-ray absorptiometry
IADL: instrumental activities of daily living
OR: odds ratio
RCS: restricted cubic spline
SD: standard deviations
SMI: skeletal muscle mass index
5-TCS: 5-time chair stand
95%CI: 95% confidence intervals

## Supplementary Material

supplementary material
