# Supplementary material for: Abnormal sleep duration is associated with sarcopenia in older Chinese people: A large retrospective cross-sectional study
Source: Open Med (Wars). 2024 Apr 1;19(1):20240938. doi: 10.1515/med-2024-0938 (PMC10998674; doi:10.1515/med-2024-0938)
Supplement: supplementary material [file med-2024-0938-sm.pdf]

Supplementary material

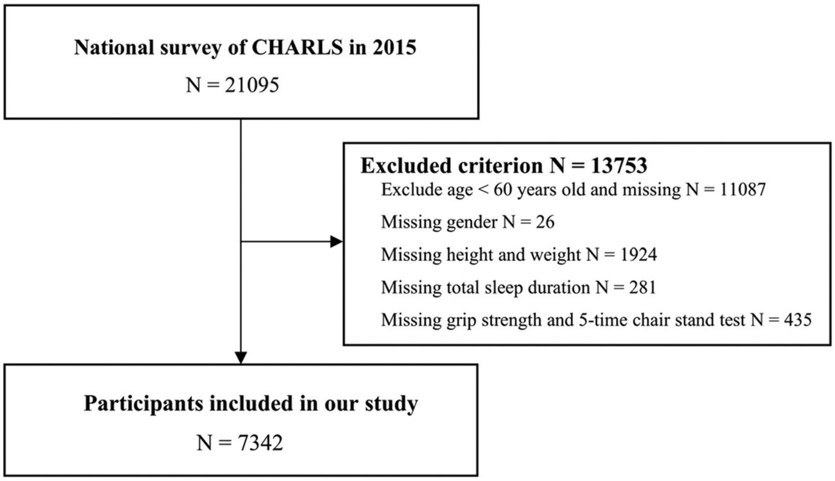

Figure S1: Flow-chart of the selection of study participants.

Table S1: Associations between the residential area difference of sarcopenia and total sleep duration by logistic regression models

Outcome variable	Analytic model	Urban			Rural		
		Short OR (95%CI)	Medium	Long OR (95%CI)	Short OR (95%CI)	Medium	Long OR (95%CI)
Possible Sarcopenia	Model 1[a]	1.46 (1.10–1.93)**	Ref.	1.12 (0.84–1.50)	1.43 (1.19–1.72)***	Ref.	1.27 (1.06–1.52)**
	Model 2[b]	1.46(1.03–2.09)**	Ref.	1.13 (0.79–1.63)	1.44 (1.13–1.83)**	Ref.	1.33 (1.05–1.68)**
	Model 3[c]	1.48 (1.03–2.13)**	Ref.	1.14 (0.78–1.65)	1.41 (1.10–1.80)**	Ref.	1.28 (1.01–1.63)**
	Model 4[d]	1.24(0.80–1.92)	Ref.	1.25(0.80–1.95)	1.35 (1.00–1.81)**	Ref.	1.42 (1.06–1.91)**
Sarcopenia	Model 1[a]	2.15 (1.34–3.45)**	Ref.	1.71 (1.05–2.78)**	1.75 (1.37–2.23)***	Ref.	1.30 (1.01–1.66)**
	Model 2[b]	1.89 (1.00–3.59)	Ref.	1.81 (0.93–3.52)	1.34 (0.95–1.87)	Ref.	1.32 (0.94–1.84)
	Model 3[c]	1.93 (1.02–3.67)**	Ref.	1.88 (0.96–3.67)	1.29 (0.92–1.81)	Ref.	1.28 (0.91–1.79)
	Model 4[d]	1.49 (0.66–3.38)	Ref.	2.02 (0.85–4.79)	1.00 (0.66–1.48)	Ref.	1.29(0.86–1.93)

***: p-value < 0.001.

**: p-value < 0.05.

[a]Crude model.

[b]Adjusted for age, gender, marital status, residential area, socioeconomic status.

[c]Adjusted for age, gender, marital status, residential area, socioeconomic status, smoke, drink.

[d]Adjusted for age, gender, marital status, residential area, socioeconomic status, smoke, drink, difficulty in daily activities, difficulty in instrumental activities, BMI, co-morbidities, hypertension, diabetes, chronic kidney disease, cognitive assessment, CES-D-10 items.

[e]Ref, Reference; CI, confidence interval; OR, odds ratio.
